# Pairing mechanism in multiband superconductors

**DOI:** 10.1038/s41598-020-63608-1

**Published:** 2020-05-04

**Authors:** Wen-Min Huang, Hsiu-Hau Lin

**Affiliations:** 1 Department of Physics, National Chung Hsing University, 40227 Taichung, Taiwan; 2 Department of Physics, National Tsing Hua University, 30013 Hsinchu, Taiwan

**Keywords:** Phase transitions and critical phenomena, Superconducting properties and materials

## Abstract

It has been a long-standing puzzle why electrons with repulsive interactions can form pairs in unconventional superconductors. Here we develop an analytic solution for renormalization group analysis in multiband superconductors, which agrees with the numerical results exceedingly well. The analytic solution allows us to construct soluble effective theory and answers the pairing puzzle: electrons form pairs resonating between different bands to compensate the energy penalty for bring them together, just like the resonating chemical bonds in benzene. The analytic solutions allow us to explain the peculiar features of critical temperatures, spin uctuations in unconventional superconductors and can be generalized to cuprates where the notion of multibands is replaced by multipatches in momentum space.

## Introduction

It has been over one hundred years since Heike Kamerlingh Onnes discovered the resistance of mercury suddenly drops to zero^1^ when cooled down by liquid helium in 1911 and marked the birth of superconductivity. The exotic phenomena of superconductors remained mysterious until Bardeen, Cooper and Schrieffer (BCS)^2^ came up with a complete theory in 1957. There are two key ingredients in BCS theory. Firstly, a negatively charged electron distorts the nearby lattice formed by positively charged ions and another electron thus feels an effective attraction toward the original electron. In professional jargons, one can say that the effective attraction is mediated by electron-phonon interactions. The second key ingredient is pair formation: an electron with specific momentum and spin pairs up with another electron with opposite momentum and spin via the effective attraction. These electron pairs, referred as Cooper pairs, form a Bose-Einstein condensate in the superconducting state^3,4^.

Despite the celebrating success of BCS theory, there are other superconductors including cuprates^5–7^, heavy-fermion compounds^8–10^, organic superconductors^11^, iron-based superconductors^12,13^ and recently found superconducting graphene superlattices^14^, remaining queer^15,16^ and unexplained by the electron-phonon interactions alone. It is generally believed that the Coulomb interaction between electrons may be responsible for the emergent superconductivity^4^. However, there is a huge mismatch of the energy scales – the critical temperature is much smaller than the bare electronic interactions. Perhaps the most intriguing puzzle for these unconventional superconductors is the pairing mechanism: what is the glue to pair up electrons from mutual repulsive interactions? The experimental evidences seem to suggest that pairing in the unconventional superconductors is not due to electron-phonon interactions^13^. Due to strong magnetic correlations^17–20^ in these materials, it is proposed that spin fluctuations^21–27^ may play the role of glue to pair electrons up. The renormalization-group (RG) studies^3^,^26–29^ seems to indicate that the antiferromagnetic spin fluctuations may be the cause of unconventional superconductivity in iron-based materials.

In this Article, we investigate the pairing mechanism in multiband iron-based superconductors by RG analysis. As in previous RG studies^3,4,26,30–32^, we integrate out quantum fluctuations at shorter length scales within the one-loop approximation and seek for the ground state in the low-energy limit. Because the RG equations are intrinsically non-linear, numerical analysis is already challenging, rendering simple understanding beneath the messy numerics inaccessible in most cases. We adapt the classification scheme of relevant couplings developed in previous works^33–35^ to overcome the challenge and analyze how superconductivity emerges in RG transformations.

In addition, we make use of the scaling relation to bridge the results obtained by RG analysis and those in the conventional mean-field theory. While it is well known that the mean-field theory provides a self-consistent description for various physical quantities, it is less familiar to the scientific community that the mean-field theory also lays out the RG equations for its parameters. For example, for the generalized multiband BCS Hamiltonian, the vanilla version of the mean-field theory is characterized by two important parameters: *g* and *g*_⊥_, standing for intraband and interband pair hopping. The functional form of the gap function computed within the mean-field theory dictates the RG equations for *g* and *g*_⊥_. In fact, these RG equations derived from the mean-field theory are so simple so that analytic solutions can be found easily.

Here comes the surprise. For multiband iron-based superconductors, we find the RG flows are well captured by simple analytic solutions for intraband and the interband pairing hopping derived from the mean-field theory. The solutions are elegant and simple enough for us to reveal the pairing mechanism in multiband superconductors.

Because the electronic interactions are repulsive in nature, both couplings *g* and *g*_⊥_ are positive before RG transformations. However, it turns out that there is no need to search for attractive glues to pair electrons up anymore. Through interband pair hopping *g*_⊥_, electron pairs hop between different bands to lower the energy, compensating the penalty for pair formation due to repulsion. Our findings are consistent with the previous studies^36,37^. The pairing mechanism in multiband superconductors is resonating pair hopping between different bands, just like the resonating chemical bonds in benzene. In fact, the effective theory we found is consistent with the previous mean-field theories for multiband superconductors in the interband-dominated regime^38–44^.

However, the RG analysis makes stronger predictions than the mean-field theories and sews several different phenomena into one coherent picture. Let us explain the major findings without digging into the technical details. The binding energy for Cooper pair formation is dictated by a small parameter $\delta =g-|{g}_{\perp }|$. As long as the interband pair hopping is larger than the intraband, Cooper pairs resonating between different bands become stable despite of the repulsive intraband interaction. Meanwhile, it is shown that the interband pair hopping also gives rise to spin fluctuations at the nesting vector which connects the dominant Fermi surfaces. The picture of resonating Cooper pairs between different bands thus provides a coherent understanding of several different phenomena: (1) Electrons can form pairs despite of mutual repulsive interactions. The onset of superconductivity can be determined by numerical RG. (2) Though the coupling strengths of *g* and *g*_⊥_ are large, the binding energy of Cooper pairs (and thus the critical temperature) is determined by their difference *δ*, which is one order smaller than the bare couplings as detailed later. (3) The interband pair hopping not only explains why strong magnetic fluctuations often show up in unconventional superconductors but also pins down at what momentum the spin fluctuations should appear.

The difference between pairing mechanism for multiband superconductors and that for the conventional BCS theory lies in the presence of interband pair hopping. In its absence, the binding energy is solely determined by *δ* = *g*, just the intraband pair hopping. Thus, for electrons to pair up, one needs some sort of attractive interactions (*g* < 0). Besides, the binding energy is of the same order of the effective attraction. But the interband pair hopping completely changes the story: attractive interaction is no longer a necessity. Despite of repulsive interactions, as long as the interaction profile produces a larger interband pair hopping, Cooper pairs form and superconductivity sets in. The interband pair hopping, driving unconventional superconductivity in the presence of repulsion, provides natural explanations, not only for the spin fluctuations at the momentum connecting the Fermi surfaces, but also for the mismatched energy scales of the critical temperature and the bare interaction strength.

## Results

To put our feet on the firm ground, we choose iron-based superconductor as a demonstrating example. In the following, we elaborate the numerical and analytical details how the above conclusions are achieved. It is worth emphasizing that most conclusions are not limited to iron-based superconductors. Generalization of our theory will be discussed at later paragraphs.

### RG for Multiband Superconductor

The kinetic energy in iron-based superconductors is described by a five-orbital tight-binding model with appropriate hopping matrix elements^45,46^. The mutual repulsion between electrons is model by the simplest on-site interactions, containing intra-orbital repulsion *U*_1_, inter-orbital repulsion *U*_2_ and the Hund’s coupling *J*_*H*_ between different orbitals. Detail descriptions of the model can be found in Methods.

The above Hamiltonian has been studied by functional renormalization group (fRG) method, which shows that the ground state is a superconductor with sign-reversed *s*_±_ pairing symmetry^30,31^. Due to the *s*-wave symmetry, it is enough to sample one point on each Fermi surfaces for qualitative understanding. It turns out that this simplification is equivalent to put the iron-based superconductor on the four-leg ladder with periodic boundary conditions along the *x* direction. It will become clear later that this geometry does not change the ground state but simplify the algebra significantly. Later, we will come back to two-dimensional calculations and reaffirm the validity of our major claims.

**Figure 1:**
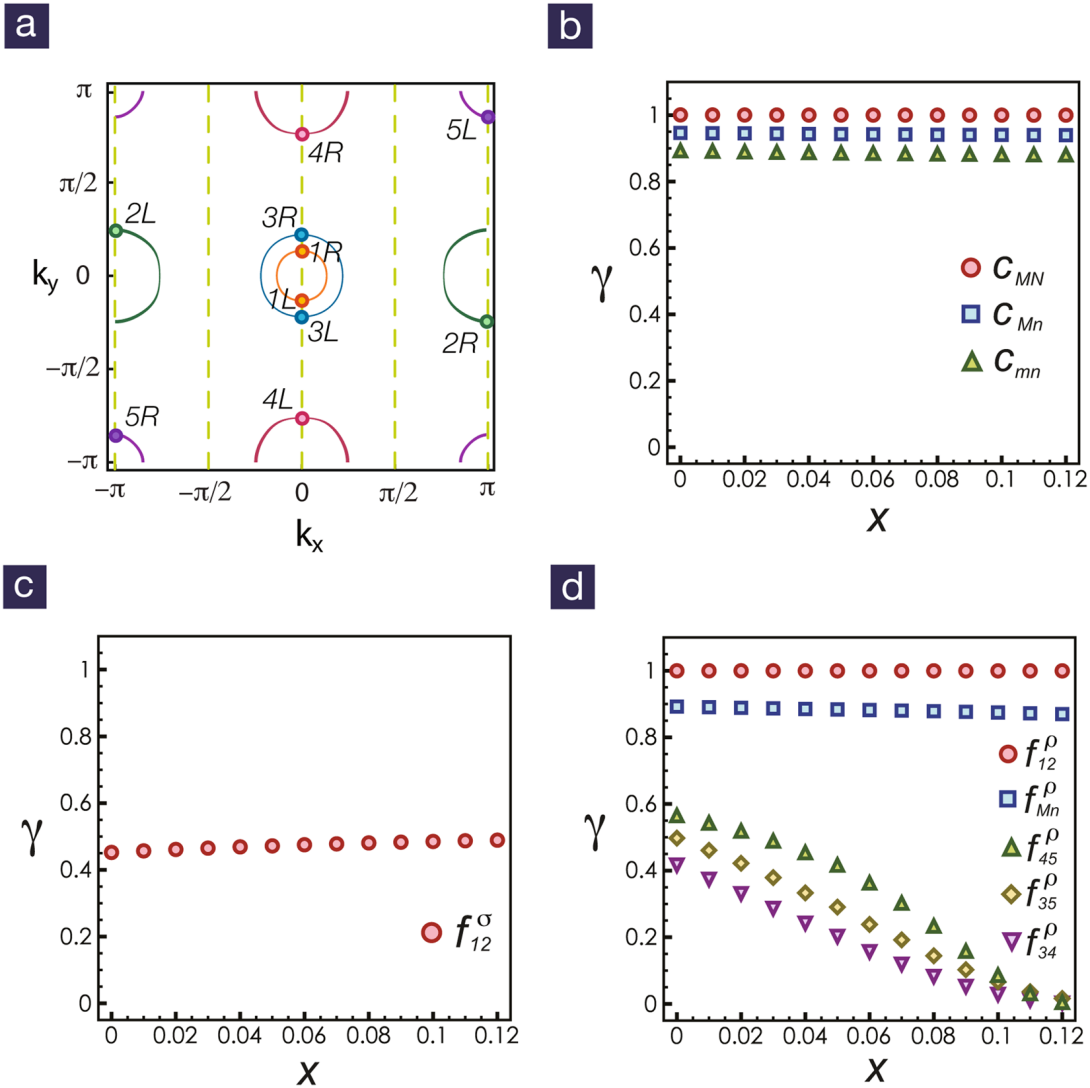
Fermi surfaces for iron-based superconductor in ladder geometry. (**a**) The Fermi surfaces from the five-orbital tight-binding Hamiltonian at electron doping *x* = 0.1. The momentum *k*_*x*_ is quantized due to the ladder geometry, reducing the five active bands to five pairs of Fermi points. (**b**) The Cooper scattering between different bands can be classified by the RG exponents. For the dominant bands (*M*, *N* = 1, 2), the exponents of ${c}_{Mn}^{\sigma },{c}_{Mn}^{\rho }$ equal 1. For the subdominant bands (*m*, *n* = 3, 4, 5), the exponents for ${c}_{mn}^{\sigma },{c}_{mn}^{\rho }$ are 0.89. The Cooper scattering between the dominant and the subdominant bands ${c}_{Mn}^{\sigma },{c}_{Mn}^{\rho }$ have RG exponents roughly 0.94. (**c**) For forward scattering in the spin sector ${f}_{12}^{\sigma }$, the exponent is about 0.45, significantly smaller than those for Cooper scattering. (**d**) In the charge sector, the exponent for ${f}_{12}^{\rho }$ is 1 and that for ${f}_{Mn}^{\rho }$ is around 0.89. All other exponents for ${f}_{34}^{\rho }$, ${f}_{35}^{\rho }$ and ${f}_{45}^{\rho }$ are significantly smaller and decrease to zero upon further electron doping. The interaction profile chosen for these RG flows is *U*_1_ = 2.8, *U*_2_ = 1.4 and *J*_*H*_ = 0.7.

In weak coupling, the relevant degrees of freedom can be labeled by the quantized momentum *k*_*x*_ and the chirality *R*/*L* as shown in Fig. 1. Following the standard field-theory description^47–49^, the allowed interactions are Cooper scattering ${c}_{ij}^{\sigma },{c}_{ij}^{\rho }$ and forward scattering ${f}_{ij}^{\sigma },{f}_{ij}^{\rho }$ between different bands, where *σ*, *ρ* denote the spin and charge channels respectively. The RG equations for all couplings are given explicitly in Methods. Though the RG equations can be written down explicitly, the solutions are still too complicated. With *l* = In(Λ_0_/Λ) being the logarithm of the ratio between bare energy cutoff Λ_0_ and the running cutoff Λ, we integrate the coupled differential equations numerically, and elaborate the details in Methods. We find all couplings are captured by the scaling Ansatz^33–35^,

1 $${g}_{i}(l)\approx \frac{{G}_{i}}{{(l-{l}_{d})}^{{\gamma }_{i}}},$$

where *g*_*i*_ are generally referred to the Cooper and forward scatterings mentioned above, *G*_*i*_ is an order one constant and 0 ≤ *γ*_*i*_ ≤ 1. The scaling exponent *γ*_*i*_ help us to build the hierarchy of all relevant couplings without ambiguity. For example, starting with *U*_1_ = 2.8 eV, *U*_2_ = 1.4 eV and *J*_*H*_ = 0.7 eV, the RG exponents for all couplings are shown in Fig. 1 with the electron doping range from *x* = 0 to *x* = 0.12.

The couplings with *γ*_i_ = 1 are the most dominant, including intraband Cooper scattering ${c}_{11}^{\rho /\sigma },{c}_{22}^{\rho /\sigma } < 0$ and the interband ones ${c}_{12}^{\rho /\sigma }\, > \,0$ between the hole pocket centered at (0,0) (band 1) and the electron pocket at $(\,\pm \pi ,\,\mathrm{0)}$ (band 2). The positive sign of the interband Cooper scattering *c*_12_ leads to the *s*_±_ pairing symmetry. Note that our RG Ansatz predicts the dominant superconducting bands with the correct pairing symmetry as obtained by the fRG method. In fact, a detail analysis including all subdominant relevant couplings lead to correct signs for all gap functions in different bands as those obtained in the fRG method. Thus, the ground state in the ladder geometry is the same as that in the two-dimensional system.

However, due to the explicit form of the RG equations for ladder geometry, the pairing mechanism in the multiband superconductor can be studied analytically. Construct the couplings, $c\equiv ({c}_{11}^{\rho }+{c}_{22}^{\rho }+{c}_{11}^{\sigma }+{c}_{22}^{\sigma }\mathrm{)/4}$ for intraband pair hopping and ${c}_{\perp }\equiv ({c}_{12}^{\rho }+{c}_{21}^{\rho }+{c}_{12}^{\sigma }+{c}_{21}^{\sigma }\mathrm{)/4}$ for interband pair hopping. It is remarkable that the RG flows obtained in numerics are well captured by the analytic solutions *g*(*l*) and *g*_⊥_(*l*),

2 $$\begin{array}{rcl}c(l)\approx g(l) & = & \frac{1}{2}\left(\frac{1}{l-{l}_{+}}+\frac{1}{l-{l}_{-}}\right),\\ {c}_{\perp }(l)\approx {g}_{\perp }(l) & = & \frac{1}{2}\left(\frac{1}{l-{l}_{+}}-\frac{1}{l-{l}_{-}}\right),\end{array}$$

where ${l}_{\pm }=-\,\mathrm{1/}[g\mathrm{(0)}\pm {g}_{\perp }\mathrm{(0)}]$ can be extracted from the numerical RG flows as the following. First of all, since the RG flows diverge at *l* = *l*_*d*_, it fixes that ${l}_{-}\,=\,\mathrm{1/[}{g}_{\perp }\mathrm{(0)}-g\mathrm{(0)]}\,=\,{l}_{d}$. Meanwhile, it is reasonable to require that $c\mathrm{(0)}+{c}_{\perp }\mathrm{(0)}\,=\,g\mathrm{(0)}+{g}_{\perp }\mathrm{(0)}$, ensuring the coupling strengths are of the same order. Because *c*(0), *c*_⊥_(*l*) are given and *l*_*d*_ is found in numerics already, the values of *g*(0) and *g*_⊥_(0) are pretty much determined.

**Figure 2:**
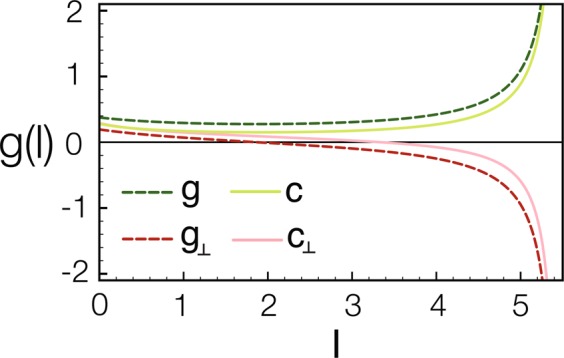
An analytic solution for the intraband and interband pair hopping. The RG flows for *c*(*l*) and *c*_⊥_(*l*) are plotted in solid lines while the solutions *g*(*l*) and *g*_⊥_(*l*) are plotted in dashed lines for comparisons. It is clear that the simple solutions capture the trends of *c*(*l*) and *c*_⊥_(*l*) rather well in the entire RG regime. As the couplings grow in RG transformation, the fluctuations are suppressed and eventually go to zero in the low-energy limit.

The above simplification is inspiring: it implies that the RG flows for the intraband and the interband pair hopping *c*(*l*) and *c*_⊥_(*l*) from integrating the whole set of nonlinear differential equations (about 50 equations for this case) can be captured by the analytic solutions given in Eq. (3). Comparison between the numerical solutions *c*(*l*), *c*_⊥_(*l*) and the analytic ones *g*(*l*), *g*_⊥_(*l*) are shown in Fig. 2, agreeing with each other exceedingly well in the whole RG regime. Thus, if we can understand the physics for the analytic solutions, we can answer the long-standing puzzle of pairing mechanism.

### Look for effective theory

To dig out the secret message behind the analytic solutions, let us switch gear to review the well-known single-band BCS Hamiltonian first. The gap function takes the form $\Delta (g)\,=\,2\Lambda \exp \mathrm{(1/}g)$, where *g* < 0 arisen from effective attraction and Λ is the Debye energy. But, it is not widely known that the RG equation for *g* can be derived from the dependence of the gap function. In the RG method, the gap function is rescaled with rescaling energy of systems in RG steps. By integrating high-energy modes, the rescaling of the gap function is described by the RG equation *d*Δ/*dl* = Δ. It is said that gap functions at different energy scales can be related by $\Delta [g\mathrm{(0)}]={e}^{-l}\Delta [g(l)]$, which is the so-called scaling argument in RG transformations^50^. Taking the derivative, *d*/*dl*, on the both sides, the RG equation reads, *dg*/*d* = −*g*^2^*l*. Therefore, if the RG flow for some system is described by the derived RG equation, its effective theory is just the single-band BCS Hamiltonian.

We can generalize the scaling argument to multiband BCS Hamiltonian as elaborated in Methods. In particular, when two bands reign interband pair hopping, the corresponding RG equations are

3 $$\begin{array}{rcl}\frac{dg}{dl} & = & -{g}^{2}-{g}_{\perp }^{2},\\ \frac{d{g}_{\perp }}{dl} & = & -2g{g}_{\perp },\end{array}$$

where *g* and *g*_⊥_ stand for intraband and interband pair hopping. The above non-linear coupled equations can be solved exactly, giving the analytic solutions we discussed previously. Therefore, the effective theory for iron-based superconductors is the multiband BCS Hamiltonian, proven by matching the RG flows together.

**Figure 3:**
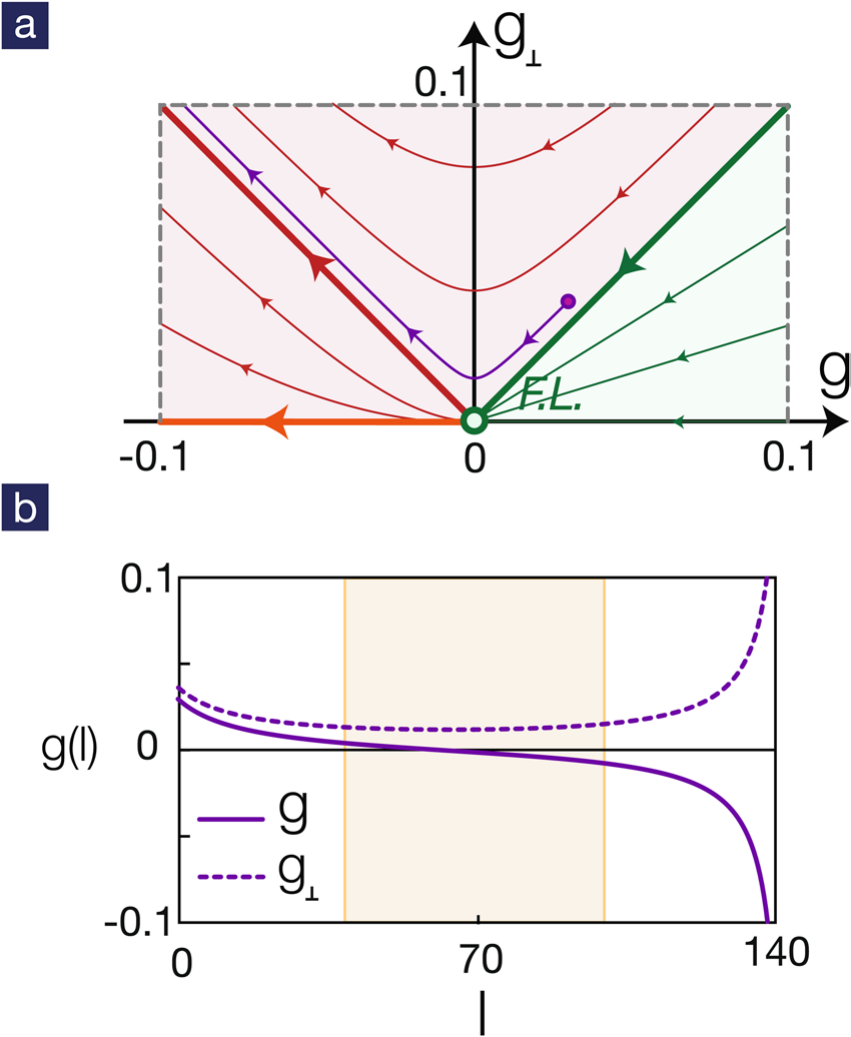
RG flows for multiband BCS Hamiltonian. (**a**) In the upper plane *g*_⊥_ > 0, the ground state is determined by the sign of the parameter $\delta =g-|{g}_{\perp }|$. For positive *δ*, it flows toward the Fermi-liquid fixed point. For negative *δ*, the ground state is a superconductor with *s*_±_ pairing symmetry. The conventional superconductor is described by the RG flows on the horizontal axis: attractive interactions are needed to drive a superconducting phase. (**b**) Typical RG flows for bare couplings close to the symmetric ray *g*_⊥_ = *g*. The corresponding trajectory on the *g* − *g*_⊥_ plane is also illustrated in part (**a**). It is a generic feature that the RG flows stay in the vicinity of the Fermi-liquid fixed point and exhibit crossover behavior in a wide range of energy scales shaded by orange areas.

RG flows for repulsive interactions are shown in Fig. 3. If the intraband pair hopping is larger than the interband one (*g* = *g*_⊥_ < 0), the ground state flows toward the Fermi-liquid fixed point and no superconductivity occurs. However, if the interband pair hopping is larger (*g* = *g*_⊥_ < 0), the ground state flows toward the superconducting phase with *s*_±_ pairing symmetry. It is worth mentioning that, if the initial couplings are close to the symmetric ray *g* = *g*_⊥_, it will flow toward the Fermi-liquid fixed point first and then turns around to the unconventional superconducting state. This means that, upon cooling down the system toward the critical temperature, it exhibits non-trivial crossover properties over a wide range of temperatures. This crossover is shown in the orange-shaded regime in Fig. 3(b) where both couplings *g* and *g*_⊥_ are small.

It is interesting to compare with the pairing instability in the absence of interband pair hopping (*g*_⊥_ = 0). As is clear in Fig. 3(a), a negative *g*(0), i.e. attractive interaction, is required to trigger the superconducting instability. For repulsive interaction *g*(0) > 0, the RG flow always bring the system to the trivial Fermi-liquid fixed point. The RG flows in the special case *g*_⊥_ = 0 give rise to the commonly accepted criterion that an effective attraction is necessary for Cooper pair formation. However, the criterion is obviously wrong as the RG flows in the upper plane is quite different from those in the horizontal axis.

The key to Cooper pair formation in the presence of interband pair hopping is characterize by the parameter

4 $$\delta =g-|{g}_{\perp }|,$$

in contrast to the intraband pair hopping *g* in conventional superconductors. The parameter *δ* offers a vivid picture for electron pairing. The penalty to form a Cooper pair within the same band is *g*. However, through interband pair hopping, a Cooper pair gains $-|{g}_{\perp }|$ benefit through resonating between different bands. Unlike the conventional superconductors, the pairing mechanism in multiband superconductors is resonating pair hopping between different bands, just like the resonating chemical bonds in benzene. Our findings are consistent with the previous discoveries of superconductivity in the interband-dominated regime^38–44^.

**Figure 4:**
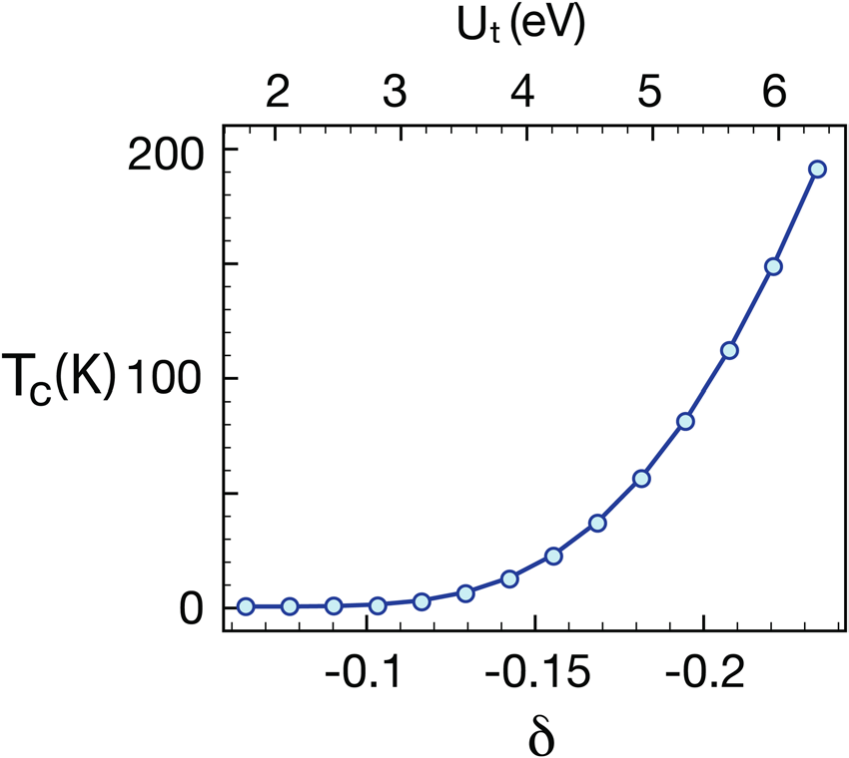
Critical temperature *T*_*c*_ versus the interaction strength. The on-site interaction strength is ${U}_{t}={U}_{1}+{U}_{2}+{J}_{H}$ with the interaction profile ${U}_{1}/{U}_{2}=2$ and ${U}_{1}/{J}_{H}=4$. The critical temperature is dictated by the small parameter *δ*, which can be extracted from numerical RG flows and is one order smaller than the bare interaction strength.

This picture leads to several significant changes of our common intuitions. First of all, the attractive interaction is no longer a necessity. As long as *δ* < 0, the generalized BCS Hamiltonian delivers a superconducting ground state. The second feature is that the critical temperature is not locked up the the energy scale of the bare interactions *g* and *g*_⊥_ anymore. Following the textbook calculations, the critical temperature is

5 $${k}_{B}{T}_{c}\simeq 1.14\,\Lambda \,{e}^{-\mathrm{1/|}\delta |},$$

where Λ is of the same order of electronic band width. Setting $\Lambda =t=1eV$, reasonable estimate for the hopping amplitude *t*, we extract *δ* from the numerical RG flows for different on-site interaction strengths ${U}_{t}={U}_{1}+{U}_{2}+{J}_{H}$ and plot the critical temperatures in Fig. 4. For the particular interaction profile we choose, ${U}_{1}/{U}_{2}=2$ and ${U}_{1}/{J}_{H}=4$, the parameter *δ* is one order of magnitude smaller that the bare coupling *U*_*t*_/*t*. This explains why the critical temperature can be so small while the bare interaction strength is large. If one chooses ${U}_{1}=2.8$, ${U}_{2}=1.4$ and ${J}_{H}=0.7$, the predicted critical temperature is about 56 K – quite a reasonable estimate.

Finally, the interband pair hopping *g*_⊥_ brings in another interesting property in unconventional superconductors. Making use of the instability analysis developed by Wang and Lee^3^, the interband pair hopping also enhances spin-density-wave (SDW) instability if the momentum ***Q*** = ***K***_1_ − ***K***_2_, connecting the two Fermi surfaces, is close to half of the reciprocal lattice vectors, i.e. ***Q*** = ***G***/2. In the case of iron-based superconductor in ladder geometry, ***Q*** = (π, 0) satisfies the above condition. Therefore, it is expected that the antiferromagnetic spin fluctuations at the momentum ***Q*** are enhanced along with unconventional superconductivity. Note that, in conventional superconductors, ***Q*** is automatically zero and no antiferromagnetic spin fluctuations are expected to grow hand-in-hand with the superconductivity.

### RG in two dimensions

**Figure 5:**
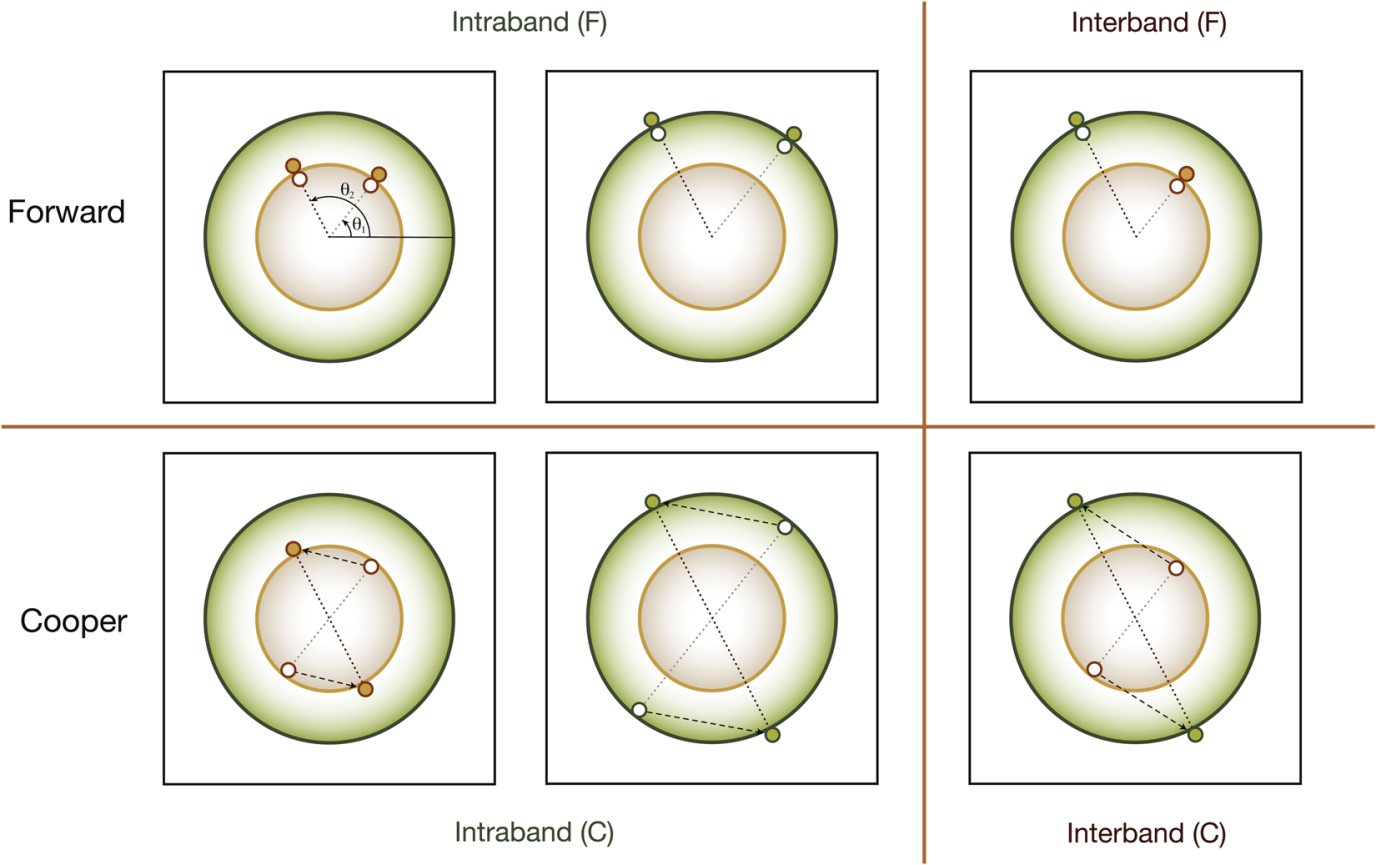
Generic 4-fermion interactions in two dimensions. The upper row represents forward scattering within the same band and that between two different bands. The lower row represents Cooper pairs hop within the the same band and between different bands.

Now that we realize the importance of interband pair hopping, we are ready to generalize Shankar’s seminal work^50^ on RG in two dimensions. In stead of following Shankar’s original approach, we follow closely the methodology developed in functional RG. Consider two circular Fermi surfaces as shown in Fig. 5. The generic 4-fermion interactions include intraband forward scattering ${F}_{PP}({\theta }_{1},{\theta }_{2})$, interband forward scattering ${F}_{P\bar{P}}({\theta }_{1},{\theta }_{2})$, intraband Cooper scattering ${C}_{PP}({\theta }_{1},{\theta }_{2})$ and interband Cooper scattering ${C}_{P\bar{P}}({\theta }_{1},{\theta }_{2})$, where $P=\mathrm{1,}\,2$ is the band index with the convention $\bar{1}=\mathrm{2,}\,\bar{2}=1$ and *θ*_*i*_ represents the angle of the corresponding momentum.

Derivations of the RG equation in two dimensions are provided in Mathods. Here we concentrate on the final results and their implications. The RG equations in two dimensions are,

6 $$\begin{array}{rcl}{\dot{C}}_{PP}({\theta }_{1},{\theta }_{2}) & = & -\,{\int }_{0}^{2\pi }\frac{d\theta }{2\pi }[{C}_{PP}({\theta }_{1},\theta ){C}_{PP}(\theta ,{\theta }_{2})+{C}_{P\bar{P}}({\theta }_{1},\theta ){C}_{P\bar{P}}(\theta ,{\theta }_{2})],\\ {\dot{C}}_{P\bar{P}}({\theta }_{1},{\theta }_{2}) & = & -\,{\int }_{0}^{2\pi }\frac{d\theta }{2\pi }[{C}_{PP}({\theta }_{1},\theta ){C}_{P\bar{P}}(\theta ,{\theta }_{2})+{C}_{P\bar{P}}({\theta }_{1},\theta ){C}_{\bar{P}\bar{P}}(\theta ,{\theta }_{2})]\mathrm{}.\end{array}$$

In the absence of interband pair hopping, we have checked that the above RG equations reduce to those derived by Shankar with just one circular Fermi surface. The general RG equations involving integration over internal angles can be simplified to the previous result. Assuming the density of states and the bare couplings are rotationally invariant, i.e. $C({\theta }_{1},{\theta }_{2})=C({\theta }_{1}-{\theta }_{2})$, the RG equations can be decoupled by the partial-wave decomposition. By identifying ${C}_{PP}\to g$ and ${C}_{P\bar{P}}\to {g}_{\perp }$, we go back to the same set of RG equations in Eq. (3) again. Therefore, our claims can be readily applied to two dimensional systems as well.

The two-pocket model presented here provides another perspective on the relation between superconductivity and antiferromagnetic spin fluctuations. In iron-based superconductors, the momentum connecting two Fermi surfaces is ${\boldsymbol{Q}}=(\pi ,\mathrm{0),}\,\mathrm{(0,}\,\pi )$ and thus the interband pair hopping enhances the antiferromagnetic spin fluctuations at these momenta. However, for the two pocket model shown in Fig. 5, both Fermi surfaces locate at the same momentum. As a consequence, though the superconductivity is still driven by the interband pair hopping, it does not come with any enhanced antiferromagnetic spin fluctuations.

## Discussion

Our previous calculations concentrate on the dominant bands in the iron-based superconductor to reveal the pairing mechanism in multiband superconductors on firm ground. But, it is reasonable to include all active bands in the generalized BCS Hamiltonian when quantitative accuracy is required. In addition, it is intriguing to check whether the fRG flows can be captured by the analytic solutions in Eq. (2) as well.

For single-band materials but with significant variations in density of states at different momenta, one should divide the Fermi surface into appropriate patches and applies the method developed here. We have carried out primitive RG analysis on cuprates by cutting the single-band Fermi surface into 16 patches (minimum to differentiate nodal and antinodal regimes with four-fold dihedral symmetry). The dominant patches locate at (π, 0) and (0, π) with sign-reversed gap functions, agreeing with the *d*-wave symmetry. It implies that pairing mechanism in cuprates arises from resonating pair hopping between antinodal regimes^51^. The interpatch pair hopping between antinodal regimes also explains the enhance spin fluctuations at (π, π). The pairing instability in the nodal regimes is only triggered when approaching close to the critical temperature. How to rigorously generalize the analysis presented here from multiband superconductors to multipatch single-band system remains an open question at this point.

We would like to emphasize that the weak-coupling RG analysis demonstrated here may work well in strong coupling if no quantum phase transition is encountered. That is to say, the generalized BCS Hamiltonian with multiple bands (or different momentum patches) may be the mother Hamiltonian for most unconventional superconductors in the low-energy limit. Even the pairing mechanism is revealed, non-trivial crossovers in the unconventional superconductors are expected at wide temperature range and a complete phenomenological description is still lacking. Finally, the pursue for higher critical temperatures should not be on seeking for stronger glues. In stead, it is time to search for optimal interaction profile which gives the largest interband pair hopping *g*_⊥_ and suppresses the intraband one *g*. It is not yet clear whether the optimal electronic interaction for different materials share a generic profile, or the answer may vary from one material to the other.

## Methods

### RG equations in ladder geometry

The band structure of the iron-based superconductor can be described by the five-orbital tight-binding model,

7 $${H}_{0}=\sum _{{\boldsymbol{p}},\sigma }\mathop{\sum }\limits_{v,w=1}^{5}{a}_{{\boldsymbol{p}}v\sigma }^{\dagger }{K}_{vw}({\boldsymbol{p}}){a}_{{\boldsymbol{p}}w\sigma },$$

where $v,w=\mathrm{1,}\,\mathrm{2,}\,\mathrm{...,}\,5$ label the five orbitals of iron, represented as $\mathrm{1:}{d}_{3{Z}^{2}-{R}^{2}}$, $\mathrm{2:}{d}_{XZ}$, $\mathrm{3:}{d}_{YZ}$, $\mathrm{4:}{d}_{{X}^{2}-{Y}^{2}}$, $\mathrm{5:}{d}_{XY}$ and $\sigma =\uparrow ,\downarrow$ is the spin index. The 5 × 5 kinetic matrix *K*_*vw*_ in the momentum space has been constructed in previous studies^45^. The Fermi surface with electron doping $x=0.1$ is illustrated in Fig. 1, which contains two hole pockets centered at (0, 0) and another hole pocket centered at (*π*, *π*) in the Brillouin zone. In addition, there are two electron pockets located at (*π*, 0) and (0, *π*) points^46^.

To investigate the correlation effects, the most general on-site interactions^30^ satisfying the SU(2) symmetry (spin rotational invariance) are

8 $$\begin{array}{rcl}{H}_{I} & = & {U}_{1}\sum _{i,v}{n}_{iv\uparrow }{n}_{iv\downarrow }+{U}_{2}\sum _{i,v < w}\sum _{\alpha \beta }{n}_{iv\alpha }{n}_{iw\beta }\\  &  & +{J}_{H}\sum _{i,v < w}[\sum _{\alpha \beta }{a}_{iv\alpha }^{\dagger }{a}_{iw\beta }^{\dagger }{a}_{iv\beta }{a}_{iw\alpha }\,+\,({a}_{iv\uparrow }^{\dagger }{a}_{iw\uparrow }{a}_{iv\downarrow }^{\dagger }{a}_{iw\downarrow }+{\rm{H}}.c\mathrm{.})],\end{array}$$

where ${n}_{iv\alpha }({\boldsymbol{r}})={a}_{iv\alpha }^{\dagger }{a}_{iv\alpha }$ and *i* runs over all lattice sites. Note that, due to the multiple orbitals, even the simplest on-site interactions contain three terms: intra-orbital repulsion *U*_1_, inter-orbital repulsion *U*_2_ and Hund’s coupling *J*_*H*_ between different orbitals.

By putting the iron-based superconductor on the four-leg ladder with periodic boundary conditions along the *x* direction, we quantize the momentum *k*_*x*_ and reduce the five active bands to five pairs of Fermi points, as show as Fig. 1a. For the ladder geometry, the RG equations for all couplings have been studied in previous literatures^33–35,47–49^, and can be written down explicitly,

9 $${\dot{c}}_{ii}^{\sigma }=-\,{({c}_{ii}^{\sigma })}^{2}-\sum _{k\ne i}\frac{{\alpha }_{ii,k}}{2}[{c}_{ik}^{\rho }{c}_{ik}^{\sigma }+{({c}_{ik}^{\sigma })}^{2}],$$

10 $${\dot{c}}_{ii}^{\rho }=-\,\sum _{k\ne i}\frac{{\alpha }_{ii,k}}{4}[{({c}_{ik}^{\rho })}^{2}+\mathrm{3(}{c}_{ik}^{\sigma }{)}^{2}],$$

11 $${\dot{c}}_{ij}^{\sigma }=-\,\sum _{k}\frac{{\alpha }_{ij,k}}{4}[{c}_{ik}^{\rho }{c}_{kj}^{\sigma }+{c}_{ik}^{\sigma }{c}_{kj}^{\rho }+2{c}_{ik}^{\sigma }{c}_{kj}^{\sigma }]+\frac{1}{2}({c}_{ij}^{\rho }{f}_{ij}^{\sigma }+{c}_{ij}^{\sigma }{f}_{ij}^{\rho }-2{c}_{ij}^{\sigma }{f}_{ij}^{\sigma }),$$

12 $${\dot{c}}_{ij}^{\rho }=-\,\sum _{k}\frac{{\alpha }_{ij,k}}{4}[{c}_{ik}^{\rho }{c}_{kj}^{\rho }+3{c}_{ik}^{\sigma }{c}_{kj}^{\sigma }]+\frac{1}{2}({c}_{ij}^{\rho }{f}_{ij}^{\rho }+3{c}_{ij}^{\sigma }{f}_{ij}^{\sigma }),$$

13 $${\dot{f}}_{ij}^{\sigma }=-\,{({f}_{ij}^{\sigma })}^{2}+\frac{1}{2}[{c}_{ij}^{\rho }{c}_{ij}^{\sigma }-{({c}_{ij}^{\sigma })}^{2}],$$

14 $${\dot{f}}_{ij}^{\rho }=\frac{1}{4}[{({c}_{ij}^{\rho })}^{2}+3({c}_{ij}^{\sigma }{)}^{2}]\mathrm{}.$$

The notation $\dot{g}\equiv dg/dl$ with $l=\,\mathrm{ln}({\Lambda }_{0}/\Lambda )$ being the logarithm of the ratio between bare energy cutoff Λ_0_ and the running cutoff Λ. The coefficient tensor ${\alpha }_{ij,k}=({v}_{i}+{v}_{k})({v}_{j}+{v}_{k})/[2{v}_{k}({v}_{i}+{v}_{j})]$ depends on the Fermi velocity *v*_*i*_ for each band. We intentionally separate the intraband and interband RG equations for clarity. Various symmetries ensure that *c*_*ij*_ = *c*_*ji*_ and *f*_*ij*_ = *f*_*ji*_. To avoid double counting, we choose *f*_*ii*_ = 0 here.

To compute the initial conditions of the RG transformation, we rewrite the field operators in the five-orbital tight-binding Hamiltonian by the chiral field decomposition^33,47–49^, i.e.

15 $${c}_{a}({\boldsymbol{r}})\simeq \frac{1}{\sqrt{4}}\sum _{{k}_{x}}\,\sum _{i}\,{e}^{i{k}_{x}x}[{e}^{i{k}_{Ri}y}{u}_{Ria}^{\ast }{\psi }_{Ri}+{e}^{-i{k}_{Li}y}{u}_{Lia}^{\ast }{\psi }_{Li}\}.$$

Here *u*_*R*/*Lia*_, *a* = 1, 2, …, 5, are the five orbital components of the Bloch wave functions associated with the *R*/*L* (or +*y*/−*y* direction) members of the *i*-th Fermi pair, and ${\psi }_{L/R}$ are chiral-field operators. After some calculation, the initial conditions of *c*^*σ*^ and *c*^*σ*^ terms can be represented as

16 $$\begin{array}{rcl}{c}_{ij}^{\sigma } & = & 2{U}_{1}\sum _{a}\,{u}_{Ria}{u}_{Rja}^{\ast }{u}_{Lia}{u}_{Lja}^{\ast }+2{U}_{2}\sum _{a\ne b}\,{u}_{Ria}{u}_{Rjb}^{\ast }{u}_{Lib}{u}_{Lja}^{\ast }\\  &  & +\,2{J}_{H}\sum _{a\ne b}\,{u}_{Ria}{u}_{Rja}^{\ast }{u}_{Lib}{u}_{Ljb}^{\ast }+2{J}_{H}\sum _{a\ne b}\,{u}_{Ria}{u}_{Rjb}^{\ast }{u}_{Lia}{u}_{Ljb}^{\ast },\end{array}$$

17 $$\begin{array}{rcl}{c}_{ij}^{\rho } & = & 2{U}_{1}\sum _{a}\,{u}_{Ria}{u}_{Rja}^{\ast }{u}_{Lia}{u}_{Lja}^{\ast }+2{U}_{2}\sum _{a\ne b}\,[2{u}_{Ria}{u}_{Ljb}^{\ast }{u}_{Lib}{u}_{Rja}^{\ast }-{u}_{Ria}{u}_{Rjb}^{\ast }{u}_{Lib}{u}_{Lja}^{\ast }]\\  &  & +2{J}_{H}\sum _{a\ne b}\,[2{u}_{Ria}{u}_{Lja}^{\ast }{u}_{Lib}{u}_{Rjb}^{\ast }-{u}_{Ria}{u}_{Rja}^{\ast }{u}_{Lib}{u}_{Ljb}^{\ast }]+2{J}_{H}\sum _{a\ne b}\,{u}_{Ria}{u}_{Rjb}^{\ast }{u}_{Lia}{u}_{Ljb}^{\ast }.\end{array}$$

With the similar computation, we also obtain the initial conditions of the forward couplings,

18 $$\begin{array}{rcl}{f}_{ij}^{\sigma } & = & 2{U}_{1}\sum _{a}\,{u}_{Ria}{u}_{Ria}^{\ast }{u}_{Lja}{u}_{Lja}^{\ast }+2{U}_{2}\sum _{a\ne b}\,{u}_{Ria}{u}_{Rib}^{\ast }{u}_{Ljb}{u}_{Lja}^{\ast }\\  &  & +2{J}_{H}\sum _{a\ne b}\,{u}_{Ria}{u}_{Ria}^{\ast }{u}_{Ljb}{u}_{Ljb}^{\ast }+2{J}_{H}\sum _{a\ne b}\,{u}_{Ria}{u}_{Rib}^{\ast }{u}_{Lja}{u}_{Ljb}^{\ast },\end{array}$$

19 $$\begin{array}{rcl}{f}_{ij}^{\rho } & = & 2{U}_{1}\sum _{a}\,{u}_{Ria}{u}_{Ria}^{\ast }{u}_{Lja}{u}_{Lja}^{\ast }+2{U}_{2}\sum _{a\ne b}\,[2{u}_{Ria}{u}_{Ljb}^{\ast }{u}_{Ljb}{u}_{Ria}^{\ast }-{u}_{Ria}{u}_{Rib}^{\ast }{u}_{Ljb}{u}_{Lja}^{\ast }]\\  &  & +2{J}_{H}\sum _{a\ne b}\,[2{u}_{Ria}{u}_{Lja}^{\ast }{u}_{Ljb}{u}_{Rib}^{\ast }-{u}_{Ria}{u}_{Ria}^{\ast }{u}_{Ljb}{u}_{Ljb}^{\ast }]+2{J}_{H}\sum _{a\ne b}\,{u}_{Ria}{u}_{Rib}^{\ast }{u}_{Lja}{u}_{Ljb}^{\ast }.\end{array}$$

We notice that the initial conditions of *g* and *g*_⊥_ of our effective theory in the main text are $g(0)=({c}_{11}^{\rho }+{c}_{22}^{\rho }+{c}_{11}^{\sigma }+{c}_{22}^{\sigma })/4$ and ${g}_{\perp }(0)=({c}_{12}^{\rho }+{c}_{21}^{\rho }+{c}_{12}^{\sigma }+{c}_{21}^{\sigma })/4$.

**Figure 6:**
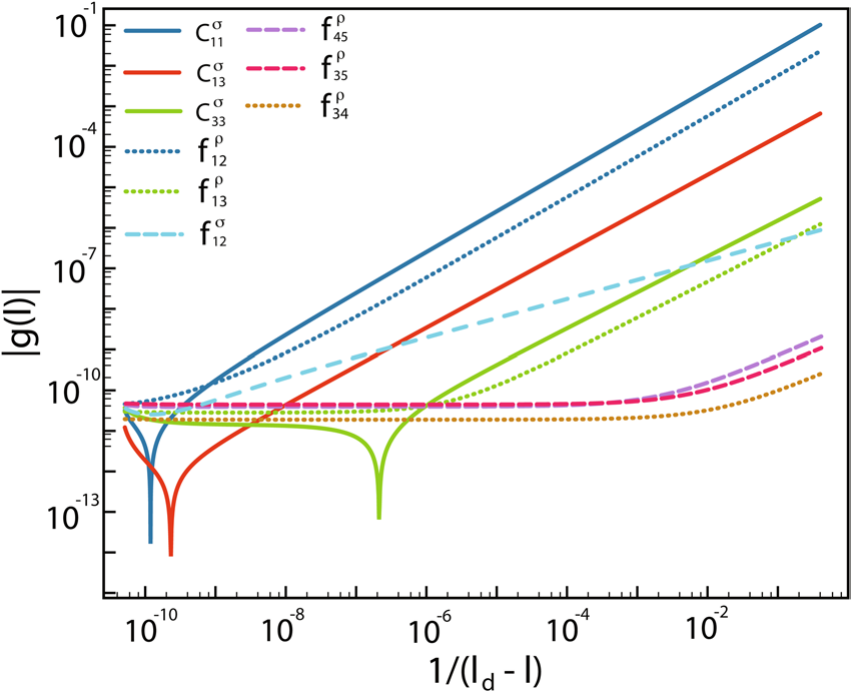
RG exponents of coupling flows. For filling factor *x* = 0.05, we plot the RG flows versus 1/(*l*_*d*_ − *l*) in the log-log plot. We capture the RG exponents by fitting the coupling constants |*g*(*l*)| from 10^−4^ to 10^−1^. The RG exponents are ${\gamma }_{{c}_{11}^{\sigma }}=1.00$, ${\gamma }_{{f}_{12}^{\rho }}=1.00$, ${\gamma }_{{c}_{13}^{\sigma }}=0.94$, ${\gamma }_{{c}_{33}^{\sigma }}=0.89$, ${\gamma }_{{f}_{13}^{\rho }}=0.89$, ${\gamma }_{{f}_{12}^{\sigma }}=0.45$, ${\gamma }_{{f}_{35}^{\rho }}=0.30$, ${\gamma }_{{f}_{45}^{\rho }}=0.42$ and ${\gamma }_{{f}_{34}^{\rho }}=0.21$.

Within the initial conditions for *U*_1_ = 2.8, *U*_2_ = 1.4 and *J*_*H*_ = 0.7, we numerically solve the RG equations to obtain the RG flows with different filling factors. We find that the coupling constants diverge at the same length scale *l*_*d*_. By showing the RG flows versus 1/(*l*_*d*_ − *l*) in the log-log plot, as illustrated in Fig. 6, we find that the hierarchy order of coupling constants can be differentiated by RG exponents in the scaling ansatz^34^. The RG exponents for different filling factors are showed in Fig. 1.

### Derivations for RG equations in two-pocket model

Following standard derivations developed in functional RG approach^52–54^, the four-fermion vertex function Γ^(4)^ is expressed as

20 $$\begin{array}{rcl}{\Gamma }^{(4)} & = & \mathop{\prod }\limits_{n=1}^{3}\,{\int }_{-\infty }^{\infty }\,\frac{d{\omega }_{n}}{{(2\pi )}^{3}}\int \,\frac{{d}^{2}{{\boldsymbol{K}}}_{n}}{{(2\pi )}^{6}}\,V({{\boldsymbol{K}}}_{1},{{\boldsymbol{K}}}_{2},{{\boldsymbol{K}}}_{3},{{\boldsymbol{K}}}_{1}+{{\boldsymbol{K}}}_{2}-{{\boldsymbol{K}}}_{3})\\  &  & {\bar{\psi }}_{\alpha }({{\boldsymbol{K}}}_{1},{\omega }_{1}){\bar{\psi }}_{\beta }({{\boldsymbol{K}}}_{2},{\omega }_{2}){\psi }_{\beta }({{\boldsymbol{K}}}_{3},{\omega }_{3}){\psi }_{\alpha }({{\boldsymbol{K}}}_{1}+{{\boldsymbol{K}}}_{2}-{{\boldsymbol{K}}}_{3},{\omega }_{1}+{\omega }_{2}-{\omega }_{3}),\end{array}$$

where *α*, *β* are spin indices, *ω* and ***K*** = (*K*_*x*_, *K*_*y*_) are labelled as the frequency and the two-dimensional momentum respectively.

**Figure 7:**
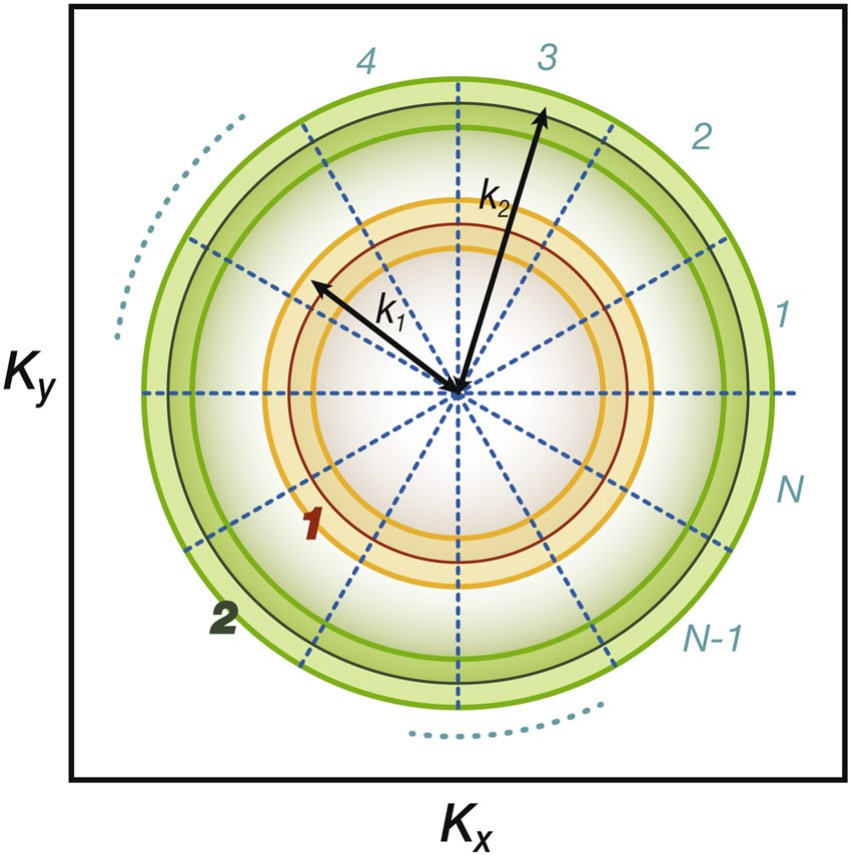
Patching the Fermi surfaces. We divide the Fermi surfaces into *N* patches. However, since the Fermi momenta are different, each patches are not of the same size. Therefore, in the derivation of RG equations, a factor related to the curvatures of Fermi surfaces will show up.

The derivation is aided by dividing the Fermi surfaces into two bands and *N* patches for each band in the vicinity of the two Fermi surfaces, determined by the energy cut-off Λ_0_, as shown in Fig. 7. Since the Fermi momenta are different, each patches are not of the same size and will give rise to a factor related to the curvatures of Fermi surfaces. Under the patching approach, we rewrite the momentum integral as

21 $$\begin{array}{rcl}\int \frac{{d}^{2}{{\boldsymbol{K}}}_{n}}{4{\pi }^{2}}\,\Theta ({\Lambda }_{0}-|E({{\boldsymbol{K}}}_{n})|) & \simeq  & \sum _{{P}_{n}\mathrm{=1,2}}\,{\int }_{|E({k}_{{P}_{n}}^{n})| < {\Lambda }_{0}}\frac{d{k}_{{P}_{n}}^{n}}{2\pi }{k}_{{P}_{n}}^{n}{\int }_{0}^{2\pi }\frac{d\theta }{2\pi },\\  & = & \mathop{\mathrm{lim}}\limits_{N\to \infty }\sum _{{P}_{n}\mathrm{=1,2}}\,{\int }_{|E({k}_{{P}_{n}}^{n})| < {\Lambda }_{0}}\frac{d{k}_{{P}_{n}}^{n}}{2\pi }{k}_{{P}_{n}}^{n}\frac{1}{N}\mathop{\sum }\limits_{i\mathrm{=1}}^{N},\end{array}$$

where momenta are constrained in the regime $({k}_{FP}-{\Lambda }_{0}/{v}_{P})\le {k}_{P}\le ({k}_{FP}+{\Lambda }_{0}/{v}_{P})$ with *k*_*FP*_ and *v*_*P*_ respectively being the Fermi momentum and Fermi velocity of the band *P*. We also approximate that the dispersion of the two Fermi pockets are independent of the angular direction. As a consequence, the tree-level RG analysis, demonstrated by Shankar^50^, shows that the only marginal couplings are the forward and Cooper scattering in this convention ${F}_{PQ}(i,j)=V(Pi,Qj,Qj,Pi)$ and ${C}_{PQ}(i,j)=V(Pi,P\bar{i},Q\bar{j},Qj)$ with the band indices $P,Q=1,2$ and $i,j=1,2,\ldots ,N$ being the patching indices. We note that the patching index $\bar{i}=i+N/2$ is represented as the time-reversal partner of the *i*-th patch. With the convention of the band indices $\bar{1}=2$ $(\bar{2}=1)$, the vertex function in the patching approach can be written as

22 $$\begin{array}{rcl}{\Gamma }^{(4)} & = & \mathop{\prod }\limits_{n=1}^{3}\,\int \,\frac{d{\omega }_{n}}{{(2\pi )}^{3}}\sum _{{P}_{n}=1,2}\,{\int }_{|E({k}_{{P}_{n}}^{n})| < {\Lambda }_{0}}\frac{d{k}_{{P}_{n}}^{n}}{{(2\pi )}^{3}}{k}_{{P}_{n}}^{n}\frac{1}{2{N}^{2}}\mathop{\sum }\limits_{i,j=1}^{N}\,{\delta }_{P,{P}_{1}}\\  &  & \{{\delta }_{{P}_{1},{P}_{3}}{\delta }_{{P}_{2},{P}_{3}}[{F}_{PP}^{s}(i,j){\bar{\psi }}_{Pi\alpha }{\bar{\psi }}_{Pj\beta }{\psi }_{Pj\beta }{\psi }_{Pi\alpha }-{F}_{PP}^{l}(i,j){\bar{\psi }}_{Pi\alpha }{\bar{\psi }}_{Pj\beta }{\psi }_{Pj\alpha }{\psi }_{Pi\beta }\\  &  & +{C}_{PP}^{s}(i,j){\bar{\psi }}_{Pi\alpha }{\bar{\psi }}_{P\bar{i}\beta }{\psi }_{P\bar{j}\beta }{\psi }_{Pj\alpha }-{C}_{PP}^{l}(i,j){\bar{\psi }}_{Pi\alpha }{\bar{\psi }}_{P\bar{i}\beta }{\psi }_{P\bar{j}\alpha }{\psi }_{Pj\beta }],\\  &  & +{\delta }_{{P}_{1},{\bar{P}}_{2}}{\delta }_{{P}_{1},{\bar{P}}_{3}}[{F}_{P\bar{P}}^{s}(i,j){\bar{\psi }}_{Pi\alpha }{\bar{\psi }}_{\bar{P}j\beta }{\psi }_{\bar{P}j\beta }{\psi }_{Pi\alpha }]\\  &  & +{\delta }_{{P}_{1},{P}_{2}}{\delta }_{{P}_{1},{\bar{P}}_{3}}[-{F}_{P\bar{P}}^{l}(i,j){\bar{\psi }}_{Pi\alpha }{\bar{\psi }}_{\bar{P}j\beta }{\psi }_{\bar{P}j\alpha }{\psi }_{Pi\beta }\\  &  & +{C}_{P\bar{P}}^{s}(i,j){\bar{\psi }}_{Pi\alpha }{\bar{\psi }}_{P\bar{i}\beta }{\psi }_{\bar{P}\bar{j}\beta }{\psi }_{\bar{P}j\alpha }-{C}_{P\bar{P}}^{l}(i,j){\bar{\psi }}_{Pi\alpha }{\bar{\psi }}_{P\bar{i}\beta }{\psi }_{\bar{P}\bar{j}\alpha }{\psi }_{\bar{P}j\beta }]\},\end{array}$$

where ${\delta }_{{P}_{n},{P}_{m}}$ is the Kronecker delta and the spin patterns are defined as ${V}^{s}={C}^{\alpha \beta \beta \alpha }$ and ${V}^{l}={V}^{\alpha \beta \alpha \beta }$ with *V* = *C*, *F*.

Under the *U*(1) and *SU*(2) symmetries, the generic form of functional RG equations for *V*^*s*^ and *V*^1^ in one-loop approximation are represented as

23 $$\begin{array}{rcl}{\dot{V}}^{s}({{\boldsymbol{K}}}_{1},{{\boldsymbol{K}}}_{2},{{\boldsymbol{K}}}_{3},{{\boldsymbol{K}}}_{4}) & = & \int \,d\kappa \\  &  & \{{L}_{pp}({\boldsymbol{K}},{{\boldsymbol{P}}}_{12}-{\boldsymbol{K}}){V}^{s}({{\boldsymbol{K}}}_{1},{{\boldsymbol{K}}}_{2},{\boldsymbol{K}},{{\boldsymbol{P}}}_{12}-{\boldsymbol{K}}){V}^{s}({{\boldsymbol{P}}}_{12}-{\boldsymbol{K}},{\boldsymbol{K}},{{\boldsymbol{K}}}_{3},{{\boldsymbol{K}}}_{4})\\  &  & +{L}_{ph}({\boldsymbol{K}},{\boldsymbol{K}}+{{\boldsymbol{Q}}}_{13}){V}^{s}({\boldsymbol{K}}+{{\boldsymbol{Q}}}_{13},{{\boldsymbol{K}}}_{2},{\boldsymbol{K}},{{\boldsymbol{K}}}_{4}){V}^{s}({\boldsymbol{K}},{{\boldsymbol{K}}}_{1},{\boldsymbol{K}}+{{\boldsymbol{Q}}}_{13},{{\boldsymbol{K}}}_{3})\\  &  & -2{L}_{ph}({\boldsymbol{K}},{\boldsymbol{K}}+{{\boldsymbol{Q}}}_{32}){V}^{l}({\boldsymbol{K}}+{{\boldsymbol{Q}}}_{32},{{\boldsymbol{K}}}_{2},{\boldsymbol{K}},{{\boldsymbol{K}}}_{3}){V}^{l}({\boldsymbol{K}},{{\boldsymbol{K}}}_{1},{\boldsymbol{K}}+{{\boldsymbol{Q}}}_{32},{{\boldsymbol{K}}}_{4})\\  &  & +{L}_{ph}({\boldsymbol{K}},{\boldsymbol{K}}+{{\boldsymbol{Q}}}_{32}){V}^{s}({\boldsymbol{K}}+{{\boldsymbol{Q}}}_{32},{{\boldsymbol{K}}}_{2},{\boldsymbol{K}},{{\boldsymbol{K}}}_{3}){V}^{l}({\boldsymbol{K}},{{\boldsymbol{K}}}_{1},{\boldsymbol{K}}+{{\boldsymbol{Q}}}_{32},{{\boldsymbol{K}}}_{4})\\  &  & +{L}_{ph}({\boldsymbol{K}},{\boldsymbol{K}}+{{\boldsymbol{Q}}}_{32}){V}^{l}({\boldsymbol{K}}+{{\boldsymbol{Q}}}_{32},{{\boldsymbol{K}}}_{2},{\boldsymbol{K}},{{\boldsymbol{K}}}_{3}){V}^{s}({\boldsymbol{K}},{{\boldsymbol{K}}}_{1},{\boldsymbol{K}}+{{\boldsymbol{Q}}}_{32},{{\boldsymbol{K}}}_{4})\},\end{array}$$

and

24 $$\begin{array}{rcl}{\dot{V}}^{l}({{\boldsymbol{K}}}_{1},{{\boldsymbol{K}}}_{2},{{\boldsymbol{K}}}_{3},{{\boldsymbol{K}}}_{4}) & = & \int \,d\kappa \\  &  & \{{L}_{pp}({\boldsymbol{K}},{{\boldsymbol{P}}}_{12}-{\boldsymbol{K}}){V}^{s}({{\boldsymbol{K}}}_{1},{{\boldsymbol{K}}}_{2},{\boldsymbol{K}},{{\boldsymbol{P}}}_{12}-{\boldsymbol{K}}){V}^{l}({{\boldsymbol{P}}}_{12}-{\boldsymbol{K}},{\boldsymbol{K}},{{\boldsymbol{K}}}_{3},{{\boldsymbol{K}}}_{4})\\  &  & -2{L}_{ph}({\boldsymbol{K}},{\boldsymbol{K}}+{{\boldsymbol{Q}}}_{13}){V}^{l}({\boldsymbol{K}}+{{\boldsymbol{Q}}}_{13},{{\boldsymbol{K}}}_{2},{\boldsymbol{K}},{{\boldsymbol{K}}}_{4}){V}^{l}({\boldsymbol{K}},{{\boldsymbol{K}}}_{1},{\boldsymbol{K}}+{{\boldsymbol{Q}}}_{13},{{\bf{K}}}_{3})\\  &  & +{L}_{ph}({\boldsymbol{K}},{\boldsymbol{K}}+{{\boldsymbol{Q}}}_{13}){V}^{s}({\boldsymbol{K}}+{{\boldsymbol{Q}}}_{13},{{\boldsymbol{K}}}_{2},{\boldsymbol{K}},{{\boldsymbol{K}}}_{4}){V}^{l}({\boldsymbol{K}},{{\boldsymbol{K}}}_{1},{\boldsymbol{K}}+{{\boldsymbol{Q}}}_{13},{{\boldsymbol{K}}}_{3})\\  &  & +{L}_{ph}({\boldsymbol{K}},{\boldsymbol{K}}+{{\boldsymbol{Q}}}_{13}){V}^{l}({\boldsymbol{K}}+{{\boldsymbol{Q}}}_{13},{{\boldsymbol{K}}}_{2},{\boldsymbol{K}},{{\boldsymbol{K}}}_{4}){V}^{s}({\boldsymbol{K}},{{\boldsymbol{K}}}_{1},{\boldsymbol{K}}+{{\boldsymbol{Q}}}_{13},{{\boldsymbol{K}}}_{3})\\  &  & +{L}_{ph}({\boldsymbol{K}},{\boldsymbol{K}}+{{\boldsymbol{Q}}}_{32}){V}^{s}({\boldsymbol{K}}+{{\boldsymbol{Q}}}_{32},{{\boldsymbol{K}}}_{2},{\boldsymbol{K}},{{\boldsymbol{K}}}_{3}){V}^{s}({\boldsymbol{K}},{{\boldsymbol{K}}}_{1},{\boldsymbol{K}}+{{\boldsymbol{Q}}}_{32},{{\boldsymbol{K}}}_{4})\},\end{array}$$

where $\int \,d\kappa ={\int }_{-\infty }^{\infty }\,\frac{d\omega }{2\pi }\int \,\frac{{d}^{2}{\boldsymbol{K}}}{{(2\pi )}^{2}}$, ${{\boldsymbol{P}}}_{ij}={{\boldsymbol{K}}}_{i}+{{\boldsymbol{K}}}_{j}$, and ${{\boldsymbol{Q}}}_{ij}={{\boldsymbol{K}}}_{i}-{{\boldsymbol{K}}}_{j}$. The *L*_*pp*_ and *L*_*ph*_ functions, depicting particle-particle and particle-hole bubble diagrams respectively, are denoted as

25 $$\begin{array}{rcl}{L}_{pp}({\boldsymbol{K}},{{\boldsymbol{P}}}_{12}-{\boldsymbol{K}}) & = & \frac{d}{dl}[G({\boldsymbol{K}},\omega ;l)G({{\boldsymbol{P}}}_{12}-{\boldsymbol{K}},-\omega ;l)]\\  & = & \frac{1}{i\omega -E({\boldsymbol{K}})}\frac{1}{-i\omega -E({{\boldsymbol{P}}}_{12}-{\boldsymbol{K}})}\frac{d}{dl}[\chi ({\boldsymbol{K}},l)\chi ({{\boldsymbol{P}}}_{12}-{\boldsymbol{K}},l)],\end{array}$$

and

26 $$\begin{array}{rcl}{L}_{ph}({\boldsymbol{K}},{\boldsymbol{K}}+{{\boldsymbol{Q}}}_{ij}) & = & \frac{d}{dl}[G({\boldsymbol{K}},\omega ;l)G({\boldsymbol{K}}+{{\boldsymbol{Q}}}_{ij},\omega ;l)]\\  & = & \frac{1}{i\omega -E({\boldsymbol{K}})}\frac{1}{i\omega -E({\boldsymbol{K}}+{{\boldsymbol{Q}}}_{ij})}\frac{d}{dl}[\chi ({\boldsymbol{K}},l)\chi ({\boldsymbol{K}}+{{\boldsymbol{Q}}}_{ij},l)],\end{array}$$

where $\chi ({\boldsymbol{K}},l)=\Theta (\Lambda -|E({\boldsymbol{K}})|)$ with running cut-off Λ is the truncating function, and the usual logarithmic derivative is represented as $\frac{d}{dl}=-\Lambda \frac{d}{d\Lambda }$.

We elaborate the computation of the first and second contributions of ${C}_{PP}^{s}(i,j)={V}^{s}(Pi,P\bar{i},P\bar{j},Pj)$ in Eq. (23), as an example to construct the full RG equations. In the patching approach, we approximate the dispersion of the *P*-th Fermi pocket as $E({k}_{P})=E({k}_{FP})+{v}_{P}q$ with $q\le |{\Lambda }_{0}/{v}_{P}|$. As a consequence, the first contribution of Eq. (23) is represented as

27 $$\begin{array}{rcl}{\dot{V}}^{s}(Pi,P\bar{i},P\bar{j},Pj) & = & \frac{1}{N}\sum _{Q=1,2}\,\mathop{\sum }\limits_{n=1}^{N}\,{\int }_{-\infty }^{\infty }\,\frac{d\omega }{2\pi }{\int }_{-{\varLambda }_{0}/{v}_{Q}}^{{\varLambda }_{0}/{v}_{Q}}\,\frac{dq}{2\pi }{k}_{FQ}{L}_{pp}(Qn,Q\bar{n})\\  &  & {V}^{s}(Pi,P\bar{i},Qn,Q\bar{n}){V}^{s}(Q\bar{n},Qn,P\bar{j},Pj)\\  & = & -\frac{1}{N}\sum _{Q=1,2}\,\mathop{\sum }\limits_{n=1}^{N}\,\frac{{k}_{FQ}}{2\pi {v}_{Q}}{V}^{l}(Pi,P\bar{i},Q\bar{n},Qn){V}^{l}(Q\bar{n},Qn,Pj,P\bar{j}),\end{array}$$

where ${\int }_{-\infty }^{\infty }\,\frac{d\omega }{2\pi }{\int }_{-{\Lambda }_{0}/{v}_{Q}}^{{\Lambda }_{0}/{v}_{Q}}\,\frac{dq}{2\pi }{L}_{pp}(Qn,Q\bar{n})=-\,1/(2\pi {v}_{Q})$ and ${V}^{s}(Pi,P\bar{i},Qn,Q\bar{n})=-\,{V}^{l}(Pi,P\bar{i},Q\bar{n},Qn)$. Meanwhile, the second contribution can also be computed,

28 $$\begin{array}{rcl}{\dot{V}}^{s}(Pi,P\bar{i},P\bar{j},Pj) & = & \frac{1}{N}\sum _{Q=1,2}\,\mathop{\sum }\limits_{n=1}^{N}\,{\int }_{-\infty }^{\infty }\,\frac{d\omega }{2\pi }{\int }_{-{\varLambda }_{0}/{v}_{Q}}^{{\varLambda }_{0}/{v}_{Q}}\,\frac{dq}{2\pi }{k}_{FQ}{L}_{ph}(Qn,Qn+Pi-P\bar{j})\\  &  & {V}^{s}(Qn+Pi-P\bar{j},P\bar{i},Qn,Pj){V}^{s}(Qn,Pi,Qn+Pi-P\bar{j},P\bar{j})\\  & = & {\delta }_{i,j}\frac{1}{N}\frac{{k}_{FP}}{2\pi {v}_{P}}{[{V}^{s}(Pi,P\bar{i},P\bar{i},Pi)]}^{2},\end{array}$$

where ${\int }_{-\infty }^{\infty }\,\frac{d\omega }{2\pi }{\int }_{-{\varLambda }_{0}/{v}_{Q}}^{{\varLambda }_{0}/{v}_{Q}}\,\frac{dq}{2\pi }{L}_{ph}(Qn,Qn+Pi-P\bar{j})={\delta }_{P,Q}{\delta }_{n,\bar{i}}{\delta }_{i,j}/(2\pi {v}_{P})$. We note that the summation of patches is constrained by the integral, which is also shown in Shankar’s single-pocket model^50^.

With the same spirit, we compute the full set of RG equations. Furthermore, to simplify the coefficients in the RG equations, we rescale the couplings as

29 $${V}_{PQ}\to \frac{\sqrt{{K}_{P}{K}_{Q}}}{\pi ({v}_{P}+{v}_{Q})}{V}_{PQ}.$$

Thus, the full set of non-trivial RG equations is represented as,

30 $${\dot{F}}_{P\bar{P}}^{s}(i,\bar{i})=\frac{1}{N}\{\gamma {[{C}_{P\bar{P}}^{s}(i,i)]}^{2}-\gamma {[{F}_{P\bar{P}}^{l}(i,\bar{i})]}^{2}\},$$

31 $$\begin{array}{rcl}{\dot{F}}_{P\bar{P}}^{l}(i,\bar{i}) & = & \frac{1}{N}\{2\gamma {C}_{P\bar{P}}^{s}(i,i){C}_{P\bar{P}}^{l}(i,i)-2\gamma {[{C}_{P\bar{P}}^{l}(i,i)]}^{2}\\  &  & -\frac{2}{\gamma }{[{F}_{P\bar{P}}^{l}(i,\bar{i})]}^{2}+\frac{1-{\gamma }^{2}}{\gamma }{F}_{P\bar{P}}^{s}(i,\bar{i}){F}_{P\bar{P}}^{l}(i,\bar{i})\},\end{array}$$

32 $$\begin{array}{rcl}{\dot{C}}_{PP}^{s}(i,j) & = & -\frac{1}{N}\mathop{\sum }\limits_{n\mathrm{=1}}^{N}\,[{C}_{PP}^{l}(i,n){C}_{PP}^{l}(n,j)+{\alpha }_{\bar{P}\bar{P},P}{C}_{P\bar{P}}^{l}(i,n){C}_{\bar{P}P}^{l}(n,j)]\\  &  & +\frac{1}{N}{\delta }_{i,j}{[{C}_{PP}^{s}(i,i)]}^{2}+\frac{1}{N}{\delta }_{j,\bar{i}}\{2{C}_{PP}^{l}(i,i){C}_{PP}^{s}(i,i)-2{[{C}_{PP}^{l}(i,i)]}^{2}\},\end{array}$$

33 $$\begin{array}{rcl}{\dot{C}}_{PP}^{l}(i,j) & = & -\frac{1}{N}\mathop{\sum }\limits_{n=1}^{N}\,[{C}_{PP}^{l}(i,n){C}_{PP}^{s}(n,j)+{\alpha }_{\bar{P}\bar{P},P}{C}_{P\bar{P}}^{l}(i,n){C}_{\bar{P}P}^{s}(n,j)]\\  &  & +\frac{1}{N}{\delta }_{i,j}\{2{C}_{PP}^{l}(i,i){C}_{PP}^{s}(i,i)-2{[{C}_{PP}^{l}(i,i)]}^{2}\}+\frac{1}{N}{\delta }_{j,\bar{i}}{[{C}_{PP}^{s}(i,i)]}^{2},\end{array}$$

34 $$\begin{array}{rcl}{\dot{C}}_{P\bar{P}}^{s}(i,j) & = & -\frac{1}{N}\mathop{\sum }\limits_{n=1}^{N}\,[{C}_{PP}^{l}(i,n){C}_{P\bar{P}}^{l}(n,j)+{C}_{P\bar{P}}^{l}(i,n){C}_{\bar{P}\bar{P}}^{l}(n,j)]\\  &  & +\frac{\gamma +1/\gamma }{N}\{{\delta }_{i,j}{C}_{P\bar{P}}^{s}(i,i){F}_{P\bar{P}}^{s}(i,\bar{i})+{\delta }_{j,\bar{i}}[{C}_{P\bar{P}}^{l}(i,i){F}_{P\bar{P}}^{s}(i,\bar{i})\\  &  & +{C}_{P\bar{P}}^{s}(i,i){F}_{P\bar{P}}^{l}(i,\bar{i})-2{C}_{P\bar{P}}^{l}(i,i){F}_{P\bar{P}}^{l}(i,\bar{i})]\},\end{array}$$

35 $$\begin{array}{rcl}{\dot{C}}_{P\bar{P}}^{l}(i,j) & = & -\frac{1}{N}\mathop{\sum }\limits_{n=1}^{N}\,[{C}_{PP}^{l}(i,n){C}_{P\bar{P}}^{s}(n,j)+{C}_{P\bar{P}}^{l}(n,l){C}_{\bar{P}\bar{P}}^{s}(n,j)]\\  &  & +\frac{\gamma +1/\gamma }{N}\{{\delta }_{j,\bar{i}}{C}_{P\bar{P}}^{s}(i,i){F}_{P\bar{P}}^{s}(i,\bar{i})+{\delta }_{i,j}[{C}_{P\bar{P}}^{l}(i,i){F}_{P\bar{P}}^{s}(i,\bar{i})\\  &  & +{C}_{P\bar{P}}^{s}(i,i){F}_{P\bar{P}}^{l}(i,\bar{i})-2{C}_{P\bar{P}}^{l}(i,i){F}_{P\bar{P}}^{l}(i,\bar{i})]\},\end{array}$$

where ${\alpha }_{ij,k}=({v}_{i}+{v}_{k})({v}_{j}+{v}_{k})/2{v}_{k}({v}_{i}+{v}_{j})$ and $\gamma =\sqrt{{k}_{FP}/{k}_{F\bar{P}}}$. Noted that because of ${C}_{PP}(i,i)={F}_{PP}(i,\bar{i})$, we choose ${F}_{PP}(i,\bar{i})=0$ to avoid double counting.

Take *N* → ∞ and the two dimensional RG emerges. Terms with 1/*N* factor but without summation vanish in the two dimensional limit. This simplification is the same as the elegant “phase-space argument” invented by Shankar^50^ before. Terms with summations smoothly evolve back into angular integral,

36 $$\mathop{\mathrm{lim}}\limits_{N\to \infty }\frac{1}{N}\mathop{\sum }\limits_{l=1}^{N}={\int }_{0}^{2\pi }\,\frac{d\theta }{2\pi },$$

because $\Delta \theta =\frac{2\pi }{N}$. Relabeling the couplings by continuous variables and rescaling of the couplings again to remove all curvature factors,

37 $${G}_{PQ}\to \sqrt{{\alpha }_{\bar{P}\bar{P},P}}\frac{\sqrt{{K}_{P}{K}_{Q}}}{\pi ({v}_{P}+{v}_{Q})}{G}_{PQ}=\frac{\sqrt{{K}_{P}{K}_{Q}}}{\pi \sqrt{{v}_{P}{v}_{Q}}}{G}_{PQ},$$

now the RG equations are in their simplest final form:

38 $${\dot{C}}_{PP}^{s}({\theta }_{1},{\theta }_{2})=-{\int }_{0}^{2\pi }\,\frac{d\theta }{2\pi }[{C}_{PP}^{l}({\theta }_{1},\theta ){C}_{PP}^{l}(\theta ,{\theta }_{2})+{C}_{P\bar{P}}^{l}({\theta }_{1},\theta ){C}_{\bar{P}P}^{l}(\theta ,{\theta }_{2})],$$

39 $${\dot{C}}_{PP}^{l}({\theta }_{1},{\theta }_{2})=-\,{\int }_{0}^{2\pi }\,\frac{d\theta }{2\pi }[{C}_{PP}^{l}({\theta }_{1},\theta ){C}_{PP}^{s}(\theta ,{\theta }_{2})+{C}_{P\bar{P}}^{l}({\theta }_{1},\theta ){C}_{\bar{P}P}^{s}(\theta ,{\theta }_{2})],$$

40 $${\dot{C}}_{P\bar{P}}^{s}({\theta }_{1},{\theta }_{2})=-\,{\int }_{0}^{2\pi }\,\frac{d\theta }{2\pi }[{C}_{PP}^{l}({\theta }_{1},\theta ){C}_{P\bar{P}}^{l}(\theta ,{\theta }_{2})+{C}_{P\bar{P}}^{l}({\theta }_{1},\theta ){C}_{\bar{P}\bar{P}}^{l}(\theta ,{\theta }_{2})],$$

41 $${\dot{C}}_{P\bar{P}}^{l}({\theta }_{1},{\theta }_{2})=-\,{\int }_{0}^{2\pi }\,\frac{d\theta }{2\pi }[{C}_{PP}^{l}({\theta }_{1},\theta ){C}_{P\bar{P}}^{s}(\theta ,{\theta }_{2})+{C}_{P\bar{P}}^{l}({\theta }_{1},\theta ){C}_{\bar{P}\bar{P}}^{s}(\theta ,{\theta }_{2})].$$

The identity ${C}^{s}({\theta }_{1},{\bar{\theta }}_{2})={C}^{s}({\theta }_{1},{\theta }_{2}\pm \pi )={C}^{l}({\theta }_{1},{\theta }_{2})=C({\theta }_{1},{\theta }_{2})$, tracing back from the definitions of these couplings, further reduces the four sets of RG equations into two sets as given in the main text. We note that all forward scattering does not flow under RG transformation in two dimensions as pointed out before^50^.

### RG equations for multiband BCS hamiltonian

We consider the standard multiband BCS Hamiltonian^55,56^.

42 $${H}_{MF}=\sum _{i,k}\,\{{\xi }_{i}({\boldsymbol{k}})[{\psi }_{i\uparrow }^{\dagger }({\boldsymbol{k}}){\psi }_{i\uparrow }({\boldsymbol{k}})+{\psi }_{i\downarrow }^{\dagger }(\,-\,{\boldsymbol{k}}){\psi }_{i\downarrow }(\,-\,{\boldsymbol{k}})]+{\Delta }_{i}({\boldsymbol{k}})[{\psi }_{i\uparrow }^{\dagger }({\boldsymbol{k}}){\psi }_{i\downarrow }^{\dagger }(\,-\,{\boldsymbol{k}})+{\rm{H}}{\rm{.c}}.]\}.$$

where ${\Delta }_{i}({\boldsymbol{k}})={\sum }_{j,{\boldsymbol{k}}{\prime} }\,{v}_{ij}({\boldsymbol{k}},{\boldsymbol{k}}{\prime} )\langle {\psi }_{j\downarrow }(-{\boldsymbol{k}}{\prime} ){\psi }_{j\uparrow }({\boldsymbol{k}}{\prime} )\rangle$ is the gap function and ${\xi }_{i}({\boldsymbol{k}})$ is the dispersion for the *i*-th band. To simplify the calculations, we drop the momentum dependence of the interaction ${v}_{ij}({\boldsymbol{k}},{\boldsymbol{k}}{\prime} )={v}_{ij}$ in the following. To comparing our RG results, we address the pairing between the two dominant bands and assume ${v}_{11}={v}_{22}$ and ${v}_{12}={v}_{21}$ to simplify the algebra. The generalized BCS Hamiltonian can be solved self-consistenly^55^ with different instabilities, depending on two parameters $g=D{v}_{11}$ and ${g}_{\perp }=D{v}_{12}$, with *D* is the density of states at the Fermi energy.

For ${g}_{\perp } > 0$ and $g-{g}_{\perp } < 0$, the generalized two-band BCS Hamiltonian gives the *s*_±_ pairing symmetry, i.e. the gap functions are related ${\Delta }_{1}=-{\Delta }_{2}={\Delta }_{{s}_{\pm }}$ and can be expressed as

43 $${\Delta }_{{s}_{\pm }}(g,{g}_{\perp })=2\Lambda \exp \left[\frac{1}{g-{g}_{\perp }}\right],$$

where Λ is the cutoff energy for the bare interaction. Similarly, for ${g}_{\perp } < 0$ and $g+{g}_{\perp } < 0$, the generalized two-band BCS Hamiltonian gives the *s*-wave pairing symmetry, i.e. the gap functions are related ${\Delta }_{1}={\Delta }_{2}={\Delta }_{s}$ and can be expressed as

44 $${\Delta }_{s}(g,{g}_{\perp })=2\Lambda \exp \left[\frac{1}{g+{g}_{\perp }}\right],$$

We connect the gap function to the RG approach, where the gap function is rescaled with rescaling energy of systems in RG steps. Taking the gap function with *s*_±_ pairing symmetry for instance, by integrating high-energy modes, the rescaling of the gap function is described by the RG equation $d{\Delta }_{{s}_{\pm }}/dl={\Delta }_{{s}_{\pm }}$^50^. It is said that gap functions at different energy scales can be related by ${\Delta }_{{s}_{\pm }}[{g}_{-}(0)]={e}^{-l}{\Delta }_{{s}_{\pm }}[{g}_{-}(l)]$, where ${g}_{-}=g-{g}_{\perp }$. Taking derivative with respect to *l* in the both side, we obtain the equation^,^,

45 $$\frac{d{g}_{-}}{dl}={\left(\frac{d{\mathrm{ln}\Delta }_{{s}_{\pm }}}{d{g}_{-}}\right)}^{-1}.$$

We combine with the gap function from generalized two-band BCS Hamiltonian Eq. (45), and end up with

46 $$\frac{dg}{dl}-\frac{d{g}_{\perp }}{dl}=-{(g-{g}_{\perp })}^{2}=-{g}^{2}-{g}_{\perp }^{2}+2g{g}_{\perp }.$$

In the same manner, the generalized two-band BCS Hamiltonian gives the *s*-wave pairing symmetry leading to the RG equation,

47 $$\frac{dg}{dl}+\frac{d{g}_{\perp }}{dl}=-{(g+{g}_{\perp })}^{2}=-{g}^{2}-{g}_{\perp }^{2}-2g{g}_{\perp }.$$

Combining the above RG equations together, we obtain Eq. (3).
